# Myositis Ossificans with Aneurysmal Bone Cystic Changes at the Thoracic Paraspinal Region: A Case Report

**DOI:** 10.3390/medicina58101452

**Published:** 2022-10-14

**Authors:** In Ho Han, You Seon Song, In Sook Lee, Dong Hwan Kim, Kyung Un Choi

**Affiliations:** 1 Department of Neurosurgery, Pusan National University Hospital, Biomedical Research Institute, Busan 49241, Korea; 2 Department of Radiology, Pusan National University Hospital, Biomedical Research Institute, Busan 49241, Korea; 3 Department of Pathology, Pusan National University Hospital, Biomedical Research Institute, Busan 49241, Korea

**Keywords:** myositis ossificans, paraspinal muscles, aneurysmal bone cyst

## Abstract

Myositis ossificans (MO) is a benign heterotopic bone formation in muscle or soft tissue. It is a self-limiting disease that is usually initiated by trauma and often occurs in the extremities of the body. Here we report a rare case of traumatic myositis ossificans caused by unusual trauma (extracorporeal shock wave therapy) at thoracic paraspinal muscles. After a needle biopsy, the lesion increased in size, and the patient’s symptoms worsened. Malignant soft tissue tumors such as osteosarcoma should be differentiated, so excision of the mass was performed. The final diagnosis was MO with aneurysmal bone cystic change. This case is a very rare form of MO that showed an unusual cause, location, clinical course, and pathologic result on follow-up. This can be an instructive case for radiologists as it is a common disease entity with unusual manifestations.

## 1. Introduction

Myositis ossificans (MO) is a benign heterotopic bone formation within skeletal muscle. Many theories have been reported concerning the etiology of MO, but minor or major traumas are the most accepted cause. So, the lesions are localized predominantly at high-risk sites of injury, such as extremities, and a paraspinal location is very rare [1]. MO is a self-limiting disease and usually decreases in size after maturation [2]. However, in some cases, it does not resolve, and the matured bone may remain and cause mechanical symptoms. In about 10–20% of MO patients, lesions result in significant functional deficits [3]. Here we report a rare case of traumatic myositis ossificans after extracorporeal shock wave therapy at thoracic paraspinal muscles that increased in size due to its aneurysmal bone cystic change.

## 2. Case Report

**Figure 1:**
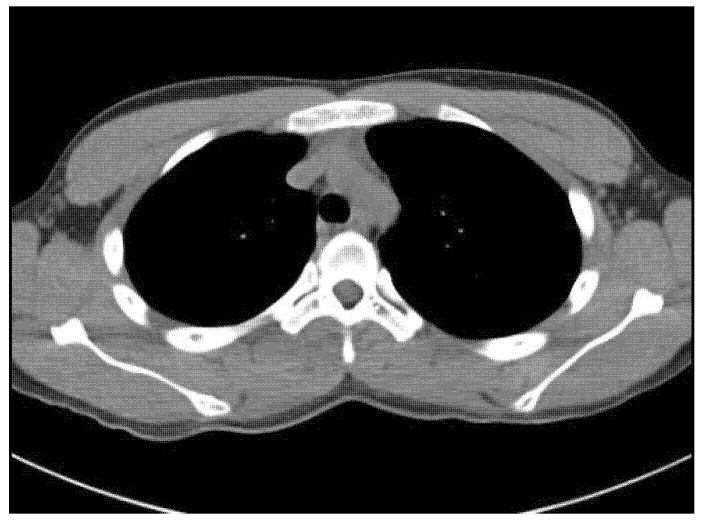
Chest CT taken at an outside hospital about 5 months prior to presentation demonstrates no abnormal mass lesion at the paraspinal area of the thoracic spine.

An 18-year-old male student came to our spine center complaining of aggravating right back pain that had started 5 months ago, along with swelling of the mid-thoracic level. At a local hospital, he underwent a chest and heart computed tomography exam for the evaluation of chest and back pain, but there were no abnormal findings on the images (Figure 1). The physician did extracorporeal shock wave therapy at his trunk for the treatment of vague pain about 6 weeks before visiting our institution. He explained that after the extracorporeal shock wave therapy, the back pain worsened, and swelling at the site of pain was found.

There was no specific finding in his medical history. However, physical examination revealed a firmly palpable, not movable, and clearly demarcated mass at the posterior mid-thoracic level with tenderness. Laboratory data were within the normal range, including C-reactive protein, erythrocyte sedimentation rate, and white blood cell counts.

**Figure 2:**
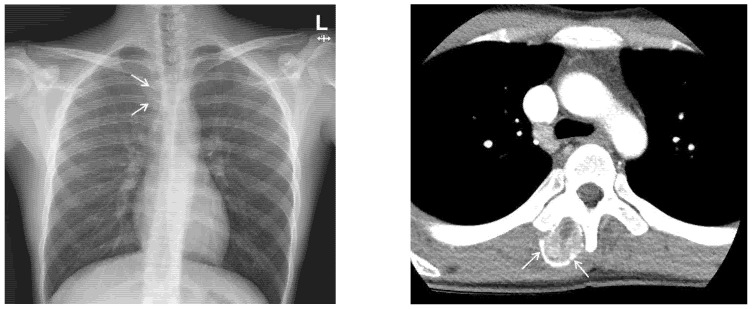
(**Left**) Chest X-ray demonstrates radiopaque mass (arrows) with rim calcification in the T4/5 paraspinal area. (**Right**) Axial CT scan shows an oval-shaped mass with peripheral rim calcification (arrows) in the right paraspinal muscle.

**Figure 3:**
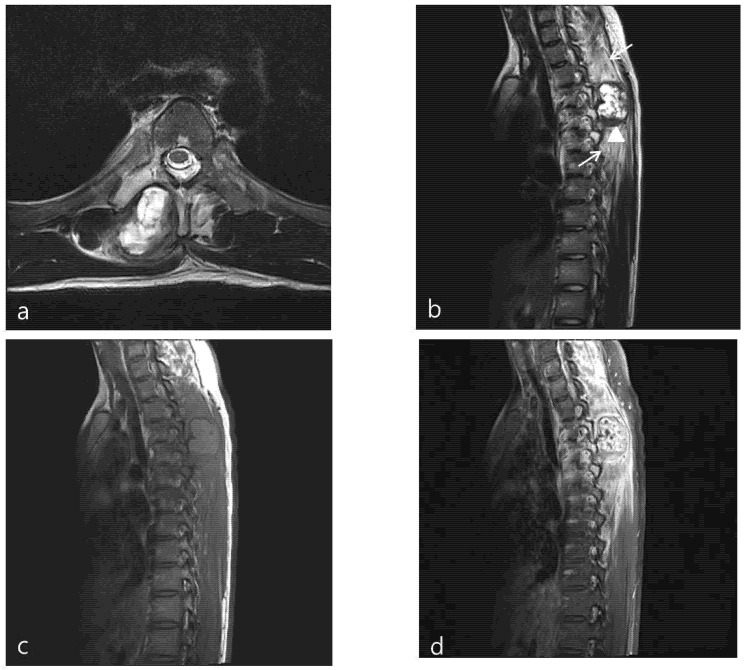
Axial (**a**) and sagittal (**b**) T2-weighted MR images show heterogeneous high SI mass in the right paraspinal area between the spinous and right transverse process of T4,5. The calcified rim (arrowhead) is shown as low SI on T2- and T1-weighted images and extensive peritumoral edema (arrows) was also noted. The mass show iso-SI on the T1-weighted image (**c**). On an enhanced (**d**) axial image, the mass shows heterogeneous enhancement.

Chest X-ray images showed a radiopaque mass with rim calcification at the T4/5 paraspinal area (Figure 2). Computed tomography images revealed an approximately 2.8 × 2.5 cm wide, 4.5 cm high oval-shaped mass in the right paraspinal muscle with peripheral rim calcification (Figure 2) that was not seen on computed tomography images 5 months prior. The cleft between the mass and the adjacent bone was visible. On magnetic resonance imaging (MRI), the mass showed a heterogeneous high signal intensity on T2WI, isointense to muscle on T1WI with a peripheral low signal intensity rim, and homogeneous enhancement. In addition, perilesional soft tissue edema at the adjacent muscle was also noted (Figure 3). With a history of suspicious trauma (extracorporeal shock wave therapy) and radiologic findings, myositis ossificans was the most suspected diagnosis.

**Figure 4:**
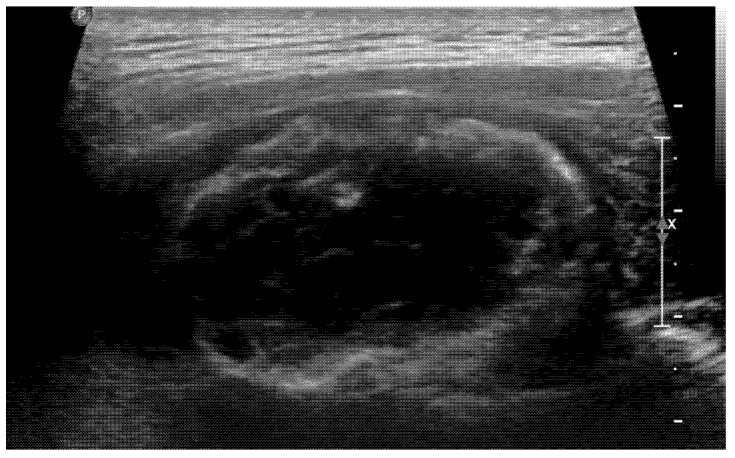
In the US, calcified peripheral rim and posterior shadowing were noted. A US-guided biopsy was performed.

**Figure 5:**
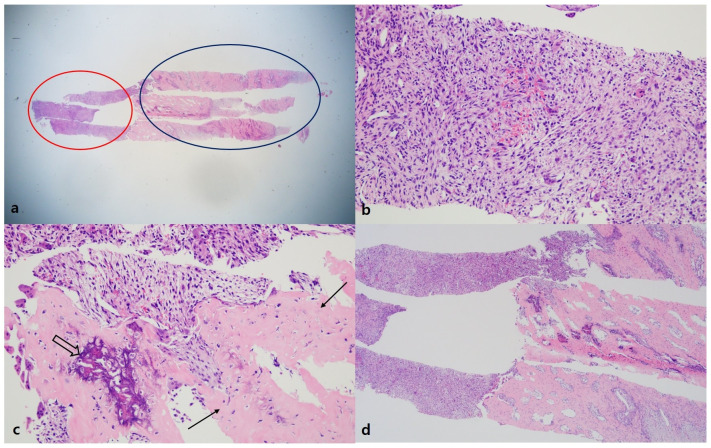
Histologic findings of biopsy (H&E stain). (**a**) On scanning view (×25), the biopsied tissue had two components, including a hypercellular area (left portion of the tissue, red-colored circle) and highly sclerotic area (right portion, blue-colored circle). (**b**) The cellular area showed ovoid to spindle-shaped cellular proliferation in edematous background. Additionally, there were extravasated erythrocytes, and the stroma had increased vascularity (×200). (**c**) The area, which was observed as hypersclerotic on scanning view, showed woven bone formation (arrows) and had mineralization (empty arrow) (×200). (**d**) The central area had a transition of the hypercellular component with woven bone formation (×40). Overall, the histologic findings were compatible with MO.

Usually, we do not perform a biopsy when MO is suspected. However, in this case, the patient’s symptoms worsened, and its location was unusual for MO, so both the clinician and patient strongly wanted to perform a biopsy. Therefore, to confirm the diagnosis and exclude the possibility of malignancy, we decided to perform a US-guided biopsy. In the US, hyperechoic peripheral rim calcification with posterior shadowing was noted (Figure 4). A US-guided needle biopsy was performed, and the pathological report confirmed myositis ossificans with zone phenomenon (Figure 5).

**Figure 6:**
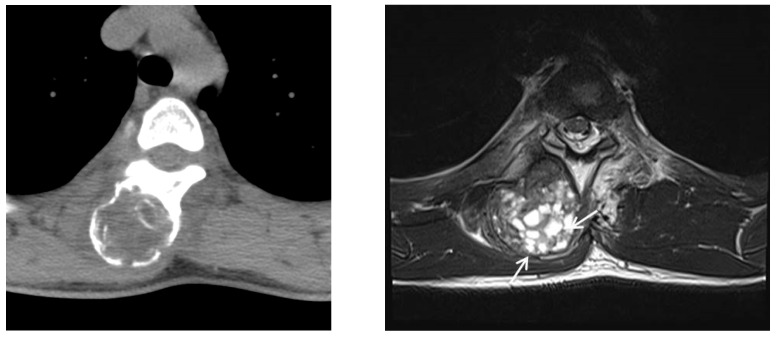
(**Left**) On follow-up CT, the paraspinal mass had enlarged compared with previous CT images. Extrinsic erosion to adjacent bone was also noted. (**Right**) Follow-up MR imaging demonstrates that multiple small cystic lesions with fluid–fluid levels (arrows) had developed in the mass, which suggested aneurysmal bone cystic change.

**Figure 7:**
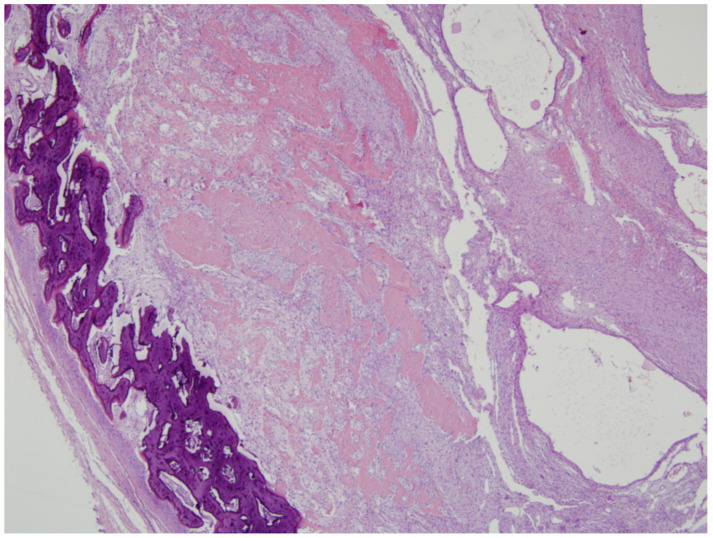
On histologic exam, the lesion contained a central cellular spindle cell area associated with immature woven bone formation and more organized mature lamellar bone at its periphery, which was consistent with myositis ossificans.

We decided to discharge and observe the patient without surgical intervention. However, follow-up thoracic spine computed tomography, 5 months after the biopsy, showed the lesion was larger than was seen on previous computed tomography (Figure 6). Moreover, the patient still complained about persistent back pain. Follow-up magnetic resonance imaging (MRI) revealed an increased size of the mass (2.8 × 2.5 × 4.5 cm → 4.3 × 4.2 × 6.3 cm) and multiple new small cystic lesions with a fluid–fluid level in the mass. Those findings suggested aneurismal bone cystic change. Furthermore, perilesional soft tissue edema of adjacent muscle was still noted (Figure 6). Because the mass did not follow the natural history of myositis ossificans, considered the possibility of a malignant tumor, such as extraskeletal osteosarcoma or parosteal osteosarcoma. So, we performed surgical excision through the posterior approach. A midline vertical incision that extended between the spinous processes of the T4 to T5 vertebrae was made. Above the left spinous process, lamina, and transverse process of T4 and T5, a well-circumscribed, gray-yellow colored, and peripheral calcified mass with gritty areas was located. The mass was somewhat fused to the left spinous process, lamina, and transverse process of T4 andT5. So, the mass was near-totally removed. On histologic exam, the lesion showed a central cellular spindle cells area associated with immature woven bone formation and more organized mature lamellar bone at the periphery that was consistent with myositis ossificans, and extensive aneurysmal bone cystic changes were also seen in the mass (Figure 7). The patient had no neurological deficits after surgical treatment. Unfortunately, the patient’s back pain had not completely disappeared, but the symptom gradually improved over a period of several months. The patient had an annual follow-up about 2 years later, and there was no evidence of recurrence of MO in the same location, and symptoms improved.

## 3. Discussion

Myositis ossificans (MO) is a benign, localized heterotopic bone formation in soft tissues, especially muscles. Although the pathophysiologic factors of myositis ossificans are not well known, an inciting event and signaling agents such as bone morphogenic protein (BMP) play an important role in the formation of heterotopic bone [1]. There are two different types of MO, including genetic form and acquired form. Several genetic diseases are associated with heterotopic ossification at multiple sites, including fibrodysplasia ossificans progressiva (FOP), progressive osseous heteroplasia (POH), and Albright’s hereditary osteodystrophy (AHO) [4]. Nongenetic forms are triggered by trauma or injury, including myositis ossificans traumatica (MOT) and neurogenic heterotopic ossification (NHO) [4]. The types of trauma include blunt trauma, penetrating wounds, fractures, dislocations, or surgical incisions [5]. Possible nontraumatic sources of provocation include infection, burns, neuromuscular disorders, hemophilia, and drug abuse [6]. There is also a report about an unusual cause of MOT, such as acupuncture [7], but there was no report that stated the cause of MOT was extracorporeal shock wave therapy. Usually, extracorporeal shock wave therapy does not cause the formation of MO, and it is even used as a treatment for post-traumatic MO [8] In our case, the site of the lesion was not the usual location of MOT. MOT can occur anywhere in the body; however, it usually occurs in the extremities, which are prone to trauma. The paraspinal muscle, as in our case, was reported as a rare site of MOT in some case reports [2,7], and most of the lesions occurred atraumatically.

The usual, natural course of MO is as follows: One or two days after trauma, a painful, tender soft tissue mass appears, which may be associated with a periosteal reaction in 7–10 days. An ossification pattern may be seen on computed tomography as early as in 2 weeks. Flocculent dens lesions arise in the mass from 2 to 6 weeks after trauma. The dense calcific areas gradually increase in size, and circumscribed peripheral calcification may be seen within a week. Maturity is reached in 5–6 months, and the lesion then shrinks [5]. Unlike the natural course of other MO, in our case, the lesion increased in size after 5 months. Because we knew the histologic result was MO, we expected the lesion would regress after 5 to 6 months. However, the lesion showed an increase in size, and the imaging represented a change inside the lesion. Sometimes, it is difficult to differentiate between MO and sarcomas since these two diseases have similar imaging findings. Łuczyńska E. et al. [9] attempted to differentiate MO from parosteal osteosarcoma, synovial sarcoma, and malignant fibrous histiocytoma. The important points for differentiation are the presence of bone erosion or destruction and the appearance of calcification. Bone erosion or destruction may be seen in sarcomas; however, it is rare in MO. In MO, calcification is usually seen in the periphery of the lesion, whereas in parosteal osteosarcoma, it is seen more centrally [9]. In our case, there was no evidence of bone erosion or destruction, and calcification was seen in the periphery of the mass. Therefore, radiologically it was more likely MO rather than sarcomas. However, considering the unusual course of progression and the few cases that were reported about the evolution to osteosarcoma from MO [10], we decided to perform an excision.

The reason for the increase in the size of the lesion was explained by the pathologic result of the excised specimen. The pathologic report represented an aneurysmal bone cystic change of the previously confirmed MO. A correlative case with our case was reported in 1992 by Amir et al. [11]. In that case, MO that occurred around the hip joint doubled in size after open biopsy, and the excised lesion was interpreted as myositis ossificans with aneurysmal bone cyst-like (ABC) changes. They emphasized that the trauma associated with the first biopsy could have initiated the formation of a hematoma in MO, and ABC-like changes in the center of the MO can be explained in terms of cystic degeneration of the hematoma. In our case, the needle biopsy can be a causative factor of hematoma formation in MO, and this could be an ABC-like change. Thus, we can learn from our case that if a lesion is thought to be MO, a needle biopsy should not be attempted to avoid the aggravation of the lesion.

In summary, this is a very rare case of MO that showed an unusual cause, location, clinical course, and pathologic result on follow-up. This can be an instructive case for radiologists as it is a common disease entity with unusual manifestations.

## Data Availability

The datasets used and/or analyzed during the current study are available from the corresponding author upon reasonable request.
